# Molecular imaging of tumour‐associated pathological biomarkers with smart nanoprobe: From “Seeing” to “Measuring”

**DOI:** 10.1002/EXP.20230070

**Published:** 2023-10-28

**Authors:** Peisen Zhang, Wenyue Li, Chuang Liu, Feng Qin, Yijie Lu, Meng Qin, Yi Hou

**Affiliations:** ^1^ College of Life Science and Technology Beijing University of Chemical Technology Beijing China; ^2^ Department of Neurosurgery and National Chengdu Center for Safety Evaluation of Drugs State Key Laboratory of Biotherapy/Collaborative Innovation Center for Biotherapy West China Hospital, Sichuan University Chengdu China; ^3^ Department of Chemistry University of Toronto Toronto Ontario Canada

**Keywords:** cancer, molecular imaging, nanoprobe, quantitative imaging, tumour microenvironment

## Abstract

Although the extraordinary progress has been made in molecular biology, the prevention of cancer remains arduous. Most solid tumours exhibit both spatial and temporal heterogeneity, which is difficult to be mimicked in vitro. Additionally, the complex biochemical and immune features of tumour microenvironment significantly affect the tumour development. Molecular imaging aims at the exploitation of tumour‐associated molecules as specific targets of customized molecular probe, thereby generating image contrast of tumour markers, and offering opportunities to non‐invasively evaluate the pathological characteristics of tumours in vivo. Particularly, there are no “standard markers” as control in clinical imaging diagnosis of individuals, so the tumour pathological characteristics‐responsive nanoprobe‐based quantitative molecular imaging, which is able to visualize and determine the accurate content values of heterogeneous distribution of pathological molecules in solid tumours, can provide criteria for cancer diagnosis. In this context, a variety of “smart” quantitative molecular imaging nanoprobes have been designed, in order to provide feasible approaches to quantitatively visualize the tumour‐associated pathological molecules in vivo. This review summarizes the recent achievements in the designs of these nanoprobes, and highlights the state‐of‐the‐art technologies in quantitative imaging of tumour‐associated pathological molecules.

## Graphical abstract

This review summarizes the recent achievements in the designs of the smart quantitative molecular imaging nanoprobes, and highlights the principle and state of the art in the quantitative visualization of tumour‐associated pathological molecules. The future prospects of nanoprobe‐based quantitative molecular imaging of cancer and the challenges facing its clinical translation are also discussed.

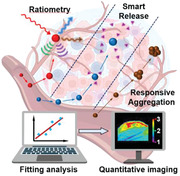

## 1 INTRODUCTION

Cancer is a kind of disease derived from the mutations of abnormal cells, which disregard the original controls of proliferation.^[^ ^1^ ^]^ The mutant cells can form the malignant tumours to invade and infiltrate the surrounding healthy tissues, and may migrate to other organs to induce metastasis.^[^ ^2^ ^]^ The tumour microenvironment where cancer cells develop, consists of non‐tumoural cells such as immune stromal or endothelial cells, and a variety of enzymes, cytokines and chemokines. This complicated cellular environment also exhibits unique physical features including lower pH, abnormal redox status, hypoxia and so on.^[^ ^3^ ^]^ The malignant behaviours of tumour cells have been demonstrated to be associated with a series of molecular events inside tumour cells or in the tumour microenvironment,^[^ ^4^ ^]^ therefore, identifying the abnormal expression of malignant pathological molecules is not only related to diagnosis and prognosis, but also has significance for therapeutic administration of cancer.^[^ ^5^ ^]^ In previous studies and clinically statistical analyses of cancer, some key characteristics that closely related to the malignancy of tumour have been demonstrated, that is, acidic tumour microenvironment, abnormal secretion of various proteases such as hyaluronidase (HAase) and matrix metalloproteinases (MMPs), excessive glutathione (GSH), hypoxia, and abnormal redox state etc.^[^ ^6^ ^]^ Nevertheless, most solid tumours display considerable complexity in vivo, leading to both spatial and temporal heterogeneity, which cannot be simply mimicked in vitro.^[^ ^7^ ^]^ In addition, the intratumoural variation for the above biomarkers may exceed the intertumoral variation, thus invalidating some population‐based prognostic studies of tumour markers.^[^ ^8^ ^]^ In the clinical scenario, it is desiderated to precisely evaluate the expression and variation of the molecular characteristics in tumour tissues dynamically.

In the past decades, medical imaging technology has become an important component of the fight against cancer, playing a crucial role in the early diagnosis, classification, and treatment response evaluation of different types of cancers. Through medical imaging, the location, size, morphology, anatomical structures, and the lymph node involvement of the solid tumours can be clearly detected. On the basis of the traditional medical imaging, in 1999, Weissleder proposed the concept of “molecular imaging”, which is aimed at the exploration of pathological molecules as the main source to generate the image contrast, rather than the nonspecific anatomical structures.^[^ ^9^ ^]^ With the customized molecular probe, the molecular imaging technology is believed to offer opportunities for non‐invasively revealing the pathological molecular characteristics of diseases in vivo. In previous publications, different molecular imaging strategies have been developed successfully to qualitatively detect the biomolecules in diseases.^[^ ^10^ ^]^ For instance, through molecular magnetic resonance imaging (MRI), the expression of human epidermal growth factor receptor‐2 (HER‐2), one of the specific receptors of tumour cells closely related to their proliferation, has been specifically imaged by targeted nanoprobes;^[^ ^11^ ^]^ through molecular PET/CT imaging, the expression level of malignance‐associated transmembrane glycoprotein CD146 can be detected by the radiolabelled specific antibodies, thereby predicting and evaluating the metastatic risk and prognosis of different types of cancers;^[^ ^12^ ^]^ through using optical molecular imaging, the intratumoural heterogeneity distribution of folate receptors in the solid tumour of colon cancer, which is closely related to the invasion and metastasis of tumour cells, can be mapped with targeted nanoprobes.^[^ ^13^ ^]^ Nevertheless, the qualitative molecular imaging may be not enough, because there are always no “standard markers” as negative controls for comparison in clinical imaging diagnosis of individuals, and the control group can only be set in laboratory experiments. From this perspective, the ultimate goal of molecular imaging is to provide quantitative information on biological molecules of interest. Only by achieving accurate and quantitative detection of the real content values of the disease‐related pathological molecules, can imaging results be used as an accepted standard and criterion for disease diagnosis.

**SCHEME 1:**
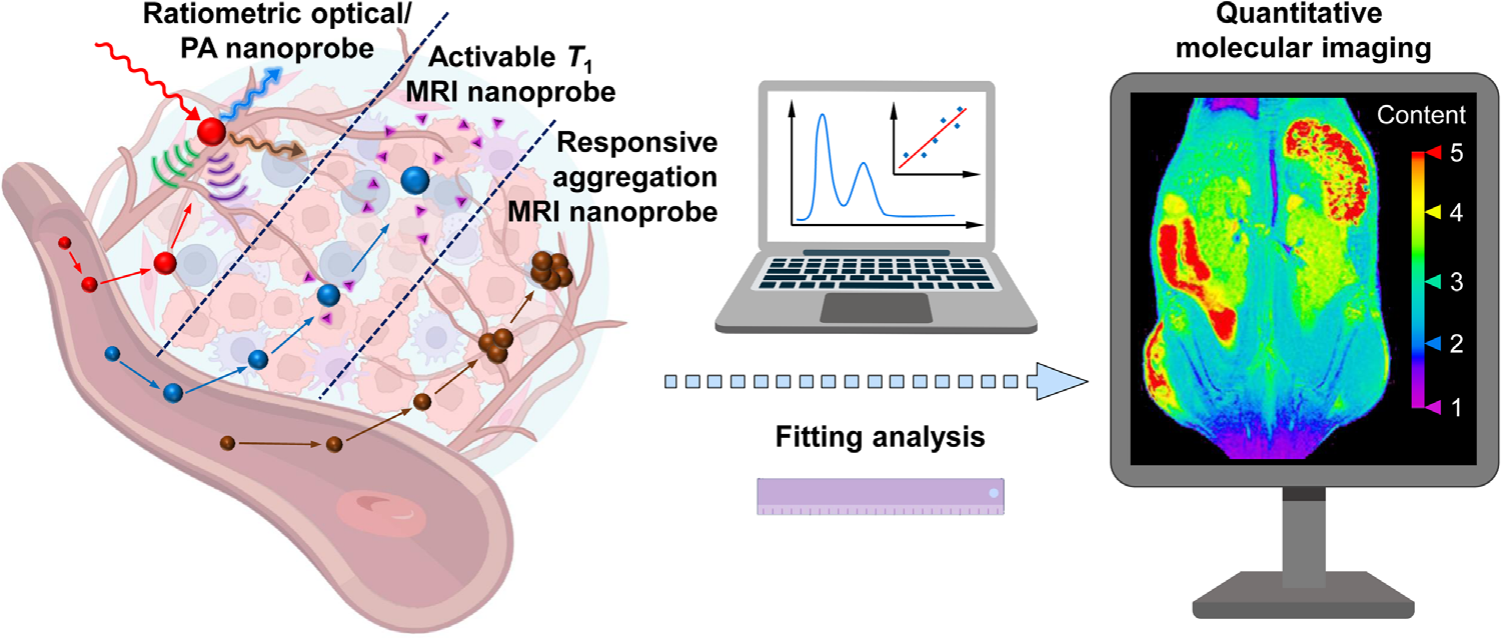
Illustration of activatable mechanism of smart quantitative nanoprobes and the quantitative visualization of pathological related biomarkers of solid tumours.

In recent years, the nanotechnology developed rapidly, and has been widely employed in the biomedical field, especially for molecular imaging.^[^ ^14^ ^]^ With respect to in vivo applications, nanomaterials exhibit diverse structure, large specific surface area and long blood half‐lives in comparison with the conventional small molecular agents. More importantly, by incorporating with biotechnology, nanomaterials can be designed and engineered to incorporate with functional or responsive moieties to construct “smart” nanoprobes. These smart nanoprobes can be activated by different stimuli inside the solid tumour to generate new signals. Through comparing the signal intensities in the different parts of tumour regions, the heterogeneity distribution of the stimuli can be reflected. In this case, taking tumour biomarkers as stimuli, the content of these biomarkers within the tumour can be visualized by the nanoprobes in vivo.^[^ ^15^ ^]^ By establishing a mathematical model, the relationship between the concentration of the biomarkers and the imaging signal or signal ratio can be expressed in a functional form. Through this function, the true concentration of biomarker in the lesions can be precisely visualized through imaging results (Scheme 1). In the current review, the state of art and novel design of smart quantitative molecular imaging nanoprobes, as well as the principles of quantitative cancer biomarkers imaging based on these nanoprobes, are summarized with the imaging modalities as the classification. These quantitative nanoprobes successfully shifted the focus of tumour molecular imaging from “seeing” to “measuring”, undoubtedly taking a technological leap forward.

## 2 QUANTITATIVE OPTICAL NANOPROBE

Optical imaging is a technology that produces the diagnosis images on the basis of the detection of the photons emitted from the fluorescent or luminescent probes in lesion sites, which is a non‐invasive and non‐ionising technology to probe cellular and molecular function in living subjects.^[^ ^16^ ^]^ In recent decades, a variety of nanoprobes have been exploited for optical imaging of cancers.^[^ ^17^ ^]^ Many of these nanoprobes are designed to specifically target cancer biomarkers, for facilitating the visualization of the spatial distribution of these molecules. Unfortunately, it is difficult for this kind of targeting nanoprobes to quantitative imaging the biomarkers, because the enhanced signal intensity in tumour region is not only derived from the active targeting behaviour of the nanoprobes, but also passive distribution of them, especially when considering the EPR effect of solid tumours.

To minimize the interference caused by passive distribution and accumulation of the nanoprobes, the ratiometric imaging strategy has been developed.^[^ ^18^ ^]^ In comparison with the absolute fluorescent intensity dependent readout, ratiometric imaging strategy is based on at least two fluorescent signals, in which one signal can be changed under the triggering of the tumour biomarker, and the other signal can be served as a reference. Through the self‐calibration, the concentration influences of nanoprobes can be eliminated. On this basis, the content of biomarkers can be precisely determined.

In 2013, Shangguan et al. reported a pH‐sensitive fluorophore, that is, 3‐amino‐1,2,4‐triazole fused 1,8‐naphthalimide (ANNA). This probe performs reversible ratiometric fluorescence under different pH.^[^ ^19^ ^]^ On top of this principle, Hou et al. constructed a pH‐sensitive ratiometric fluorescent probe by covalently conjugating ANNA to the N‐terminal of the cell‐penetrating TAT peptide. In the subsequent in vitro experiments, they demonstrated that the resultant TAT‐ANNA probe can well penetrate the tumour cell membrane to enter the cytoplasm, and monitor the intracellular pH quickly in real time. In the in vivo experiments, this imaging probe can be further employed for the semiquantitative monitoring of the intratumoural pH variation, realizing the early prediction of the pharmacodynamics of the anti‐cancer drugs in vivo.^[^ ^20^ ^]^ This study demonstrated that the molecular features of tumours revealed by molecular imaging can be referenced to determine the clinical actions during treatment.

Apart from the above semi‐quantitative pH monitoring, the responsive fluorescent dye ANNA has also been used to construct multi‐stimuli responsive nanoprobes, for achieving the simultaneous quantitative detection of different tumour pathological biomarkers. In 2015, Hou et al. designed a smart nanoprobe, which can be dually triggered by protease and pH simultaneously, in order to visualize the pH variation in the MMP‐9 highly expressed regions of solid tumours. In detail, the ANNA molecules were covalently conjugated on the Fe_3_O_4_ nanoparticles through MMP‐9 substrate peptides as the crosslinker. Owing to the Fluorescence resonance energy transfer effect between Fe_3_O_4_ and ANNA, the fluorescent signals of ANNA can be quenched by the Fe_3_O_4_ nanoparticles. However, in the MMP‐9‐rich regions of the solid tumours, the substrate peptide would be cut to subsequently release the ANNA, thereby recovering the fluorescent signals of ANNA to quantitatively map the pH values.^[^ ^21^ ^]^

**FIGURE 1:**
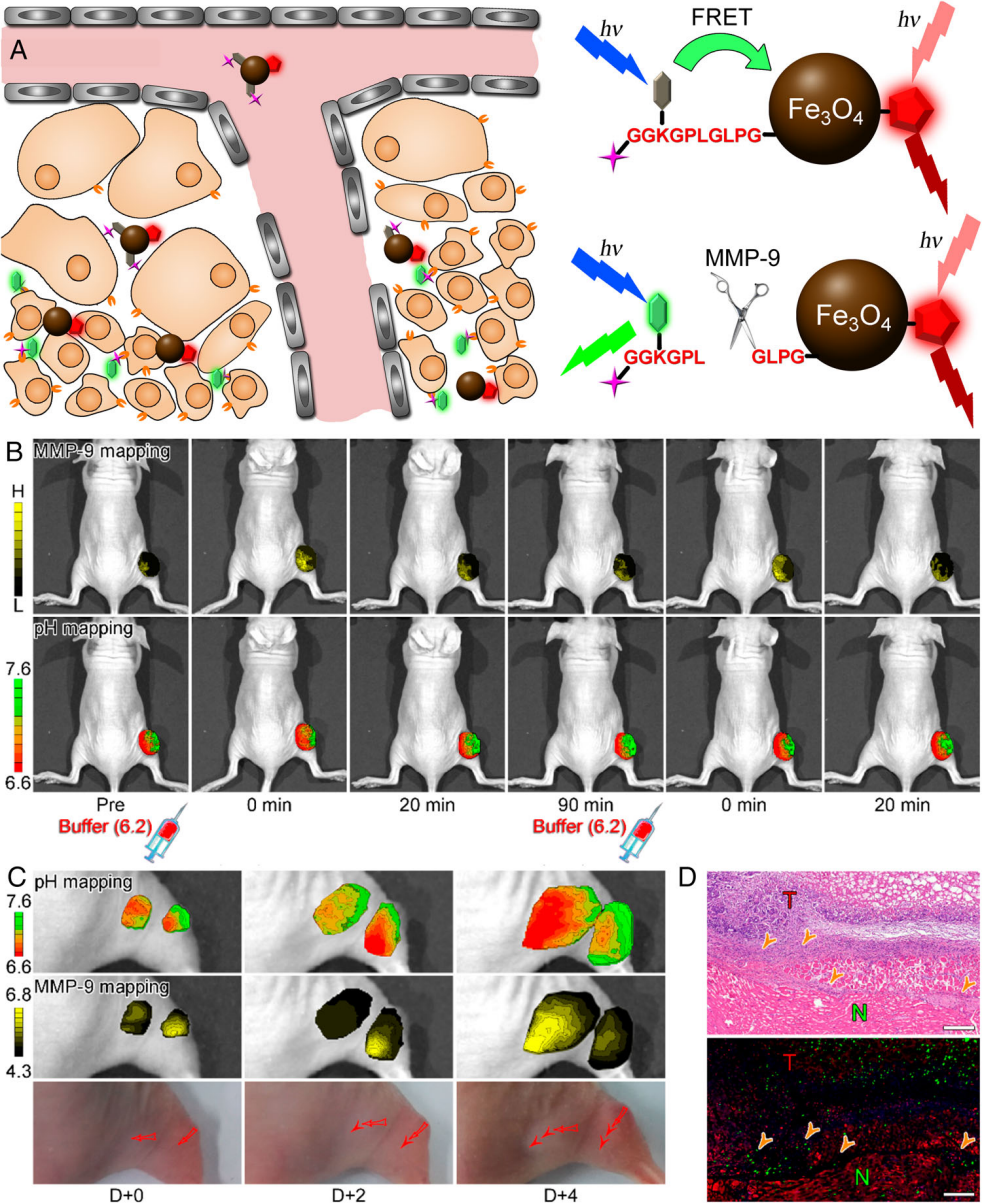
The work principle of dual‐ratiometric target‐triggered fluorescent probe and the simultaneous quantitative visualization of protease activity and pH in vivo. (A) The schematic illustration of simultaneous visualization MMP‐9 and pH with dual‐ratiometric target‐triggered fluorescent nanoprobes. (B) Variation in MMP‐9 activity in response to tumour pH adjusted upon intratumoural injection of PBS. (C) Quantified pH and MMP‐9 expression mapping of tumours obtained at D+0, D+2, and D+4. (D) Top: microscopic image of a tissue slide stained with H&E, bottom: immunofluorescence microscopic image stained for E‐cadherin (red) and MMP‐9 expression (green). The scale bars correspond to 200 μm. The orange arrows point to boundaries between healthy tissue and the tumour where MMP‐9 is highly expressed. Reproduced with permission.^[^ ^22^ ^]^ Copyright 2018, American Chemical Society.

Based on this study, in 2018, Gao et al. further introduced a pH‐independent fluorescent dye Cy5.5 on the Fe_3_O_4_ nanoparticles, which can be served as an internal reference. In this design concept, two ratiometric systems are constructed, that is, the pH ratiometry derived from two fluorescent signals of ANNA with different wavelengths, and the MMP ratiometry derived from the fluorescent of Cy5.5 and the total fluorescent signal of ANNA (Figure 1A). These dual ratiometric systems allow the quantitative visualization of pH and MMP‐9 activity simultaneously inside solid tumours in vivo. Interestingly, after the pH of solid tumours were manually adjusted through intratumoural injections of buffer solution, the quantitative imaging revealed that the activity of MMP‐9 and the pH variation in tumour tissue were well correlated with each other (Figure 1B). In addition, the nanoprobe‐based quantitative imaging also revealed that the invasion directions of tumour cells during the tumour development process is also closely related with the pH variation and MMP‐9 expression (Figure 1C,D). Through this tumour growth pattern, the direction of tumour invasion can be well predicted.^[^ ^22^ ^]^

**FIGURE 2:**
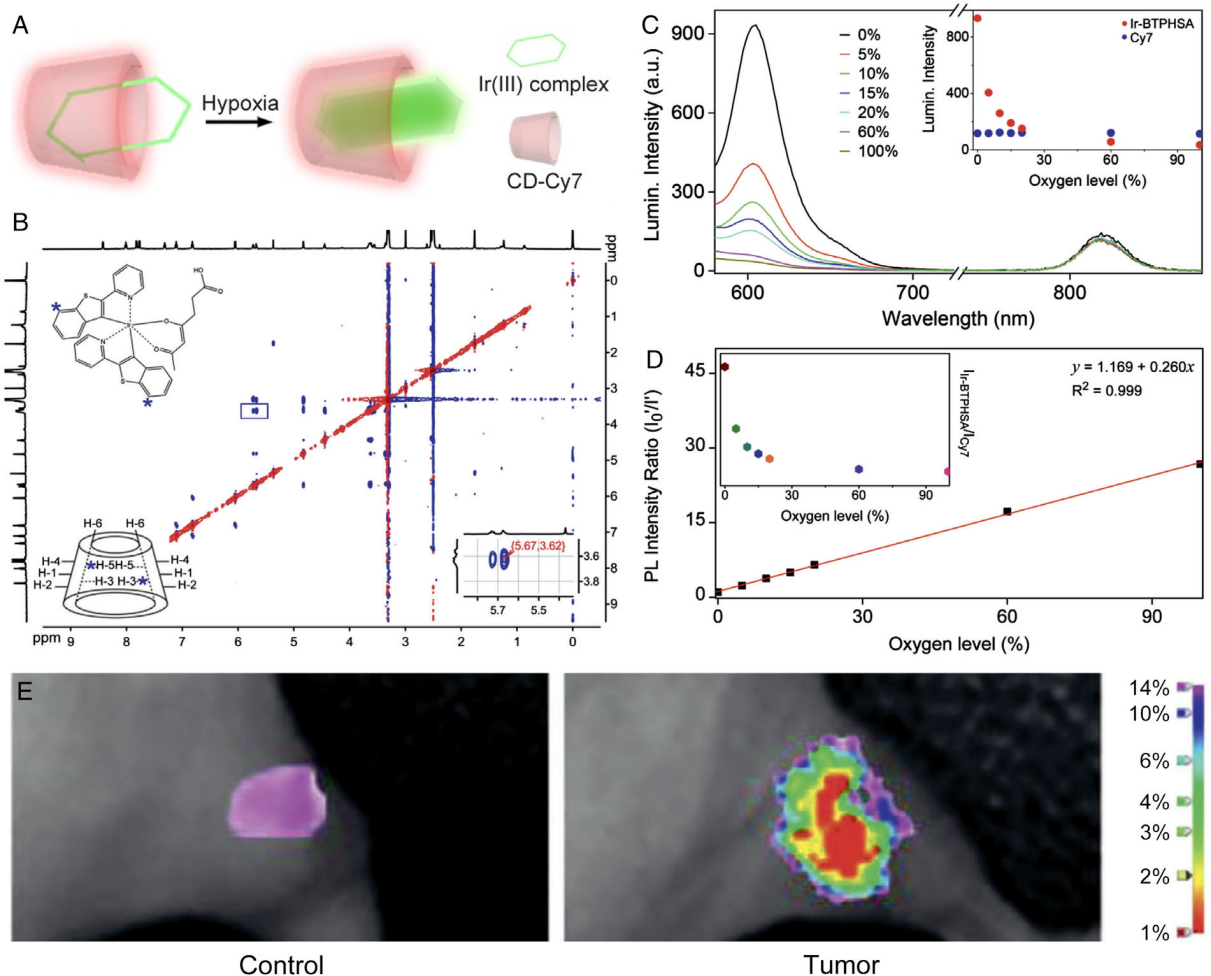
The structure, characterization of ratiometric O_2_ probe and the ratiometric mapping of tumour hypoxia in vivo. (A) The schematic illustration of cyclodextrin‐hosted Ir(III) complex. (B) 2D‐ROESY spectra of Ir‐BTPHSA/CD‐NH_2_. (C) Luminescence spectra of Ir‐BTPHSA/CD‐Cy7 recorded under various oxygen levels upon excitation at 488 and 747 nm for Ir‐BTPHSA complex and Cy7, respectively (Inset: PL intensities of Ir‐BTPHSA and Cy7 against oxygen levels), (D) a liner fitting of *I*_0_′/*I*′ against the oxygen level (*I*_0_′and *I*′ refer to PL intensities of Ir‐BTPHSA recorded at 0% oxygen and specific oxygen levels, respectively, after normalized with reference PL intensity of Cy7) (inset: normalized PL intensity of Ir‐BTPHSA against the oxygen level). (E) Oxygen level mapping of the tumour and its control site based on the normalized Ir‐BTPHSA signal and its correlation with oxygen level. Reproduced with permission.^[^ ^23^ ^]^ Copyright 2021, Wiley‐VCH.

Apart from the pH and MMP enzymes, another key microenvironmental feature of solid tumours, hypoxia, can also be quantitatively visualized through ratiometric optical imaging. For example, the fluorescence emitted by the ruthenium(II) and iridium(III) complexes could be sensitively quenched by the O_2_. Through making use of this principle, Gao et al. constructed a ratiometric O_2_ probe through hosting a hydrophobic iridium complex with red emission in β‐cyclodextrin (β‐CD) (denoted as Ir‐BTPHSA). To eliminate the influence of uneven local concentration of probe, an oxygen‐insensitive fluorophores, Cyanine7 (Cy7), was introduced and conjugated on the β‐CD, as an internal reference, to realize the ratiometric fluorescence pair with the inside Ir(III) dye for quantitatively sensing the O_2_ level. The subsequent experiments demonstrated that the probe can sensitively respond to the change of O_2_ concentration. Through a series of mathematical analysis, this probe could be employed to quantitatively reveal the hypoxia status of tumour microenvironment and determining the O_2_ level in vivo (Figure 2).^[^ ^23^ ^]^ In addition to directly detect the O_2_ in solid tumour, the abnormal expression of several hypoxia‐related biomarkers can also be selected to indirectly evaluate the tumour hypoxic status. For example, nitroreductase, a kind of enzymes that can promote the reduction of the nitro group into an amino group in nitroaromatic substrates under hypoxic conditions, has been demonstrated to be up‐regulated in hypoxic tumour cells which can serve as a hypoxia biomarker.^[^ ^24^ ^]^ On this basis, Tian et al. reported a self‐calibrated smart nanoprobe, known as Cy7‐1/PG5‐Cy5@LWHA, which can be activated to specifically detect the upregulation of nitroreductase activity in tumour cells under hypoxic condition. Specifically, an activable fluorescent reporter, Cy7‐1, which can be catalytically reduced as Cy7‐NH_2_ by nitroreductase to significantly enhance its fluorescence intensity, and a nitroreductase‐independent internal reference fluorophore, Cy5, were combined together via poly(amidoamine) dendrimer PG5. The low molecular weight hyaluronic acid (LWHA) was further employed to coat the above conjugates to endow the resultant nanoprobe with CD44 targeting ability. Through the quantitative imaging with this nanoprobe, the hypoxia in orthotopic and metastatic breast cancer of animal models can be quantitatively assessed in vivo, which have been confirmed by the immunofluorescence and qPCR assays.^[^ ^25^ ^]^

Another specific molecular feature of solid tumours, abnormal redox state, has been also visualized through quantitative optical imaging. Song et al. designed a GSH responsive two‐photon probe (TPEF‐GSH), which can be employed to quantitatively mapping GSH in the tumours of zebrafishes. The probe was composed of a fluorophore (heptamethine cyanine, Cy7) and a quenching group (p‐nitrophenyl). With the presence of GSH, the quenching group of probes can be cut to trigger the absorbance and the fluorescence of Cy7. The quantitative relationship between the GSH‐dependent fluorescent signal ratio *R*_red__/__green_ (Δ*I*_600–700_/Δ*I*_480–550_) and GSH concentrations was successfully established. With this ratiometric probe, the endogenous GSH levels of tumours in zebrafish were determined as 4.66 and 5.16 mm through injection and incubation administration routes, respectively, which is in line with the traditional cognition of GSH concentration in solid tumours (2–10 mm).^[^ ^26^ ^]^

**FIGURE 3:**
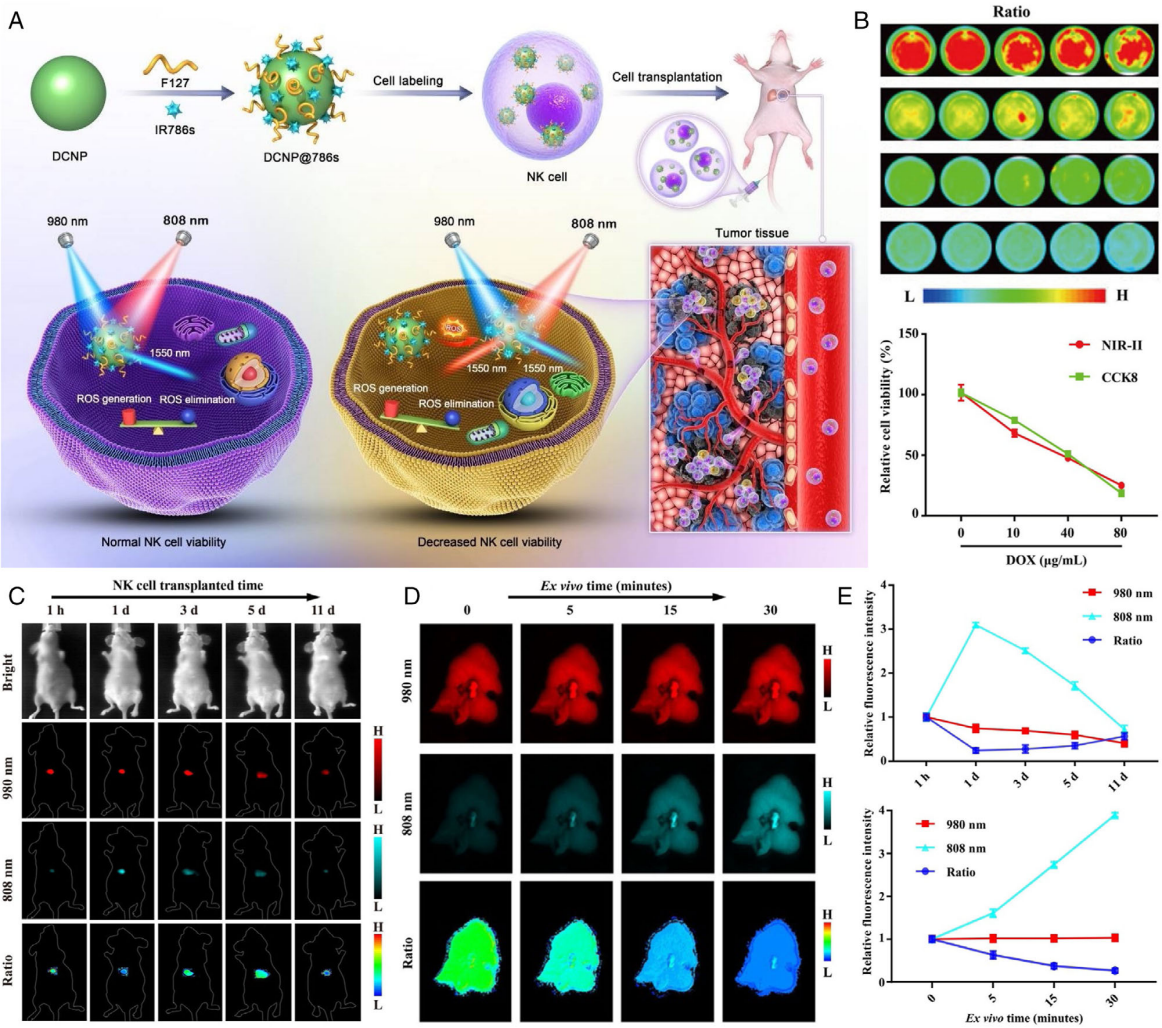
The work principle of DCNP@786s nanoprobe and the quantitatively tracking of adoptive NK cell viability in vivo. (A) Schematic illustration of DCNP@786s with ratiometric NIR‐II fluorescent signal for tracking NK cell viability in vivo. (B) The NIR‐II ratio mapping of DCNP@786s‐labeled NK cells after treatment with different concentrations of DOX for 24 h, together with the correlation of NIR‐II ratio signal and CCK8 for NK cell viability assay after treatment with different DOX doses. (C) Ratiometric NIR‐II fluorescence imaging after NK cell transplantation at different time points in vivo and (D) hepatic tissues ex vivo, together with (E) quantitative analysis of the relative signal intensities and ratios in vivo (upper) and ex vivo (lower). Reproduced with permission.^[^ ^28^ ^]^ Copyright 2021, Wiley‐VCH.

In addition to the tumour diagnosis and prognosis evaluation, the ratiometric optical quantitative imaging was also be employed to early evaluate the effectiveness of tumour immunotherapy. As known, the therapeutic efficacy of adoptive cellular immunotherapy is closely associated with the survival rate and durability of transplanted immune cells.^[^ ^27^ ^]^ However, this survival rate in vivo is difficult to assess during the treatment progress. Song et al. reported a ratiometric NIR‐II fluorescent imaging approach to real‐time quantitatively track the survival rate of the adoptive natural killer (NK) cells in vivo. The DCNP@786s nanoprobe was constructed through coating the ROS responsive NIR dye IR786s onto the surface of lanthanide‐based down‐conversion nanoparticles (DCNP). If the cell death, the excessive ROS of NK cells would degrade IR786s, reversing the quenched 1550 nm NIR‐II signal of DCNP absorbed by IR786s based on the absorption competition‐induced emission mechanism. While the fluorescent signal at 1550 nm generated through the excitation of 980 nm laser remained constant. On this basis, the quantitative correlation between the viability of NK cells, and the ratio of 1550 nm NIR signal under 980 and 808 nm excitation was established. As a result, with the quantitative imaging of NK cell viability, this study demonstrated that IL‐2, IL‐15, and IL‐21 are beneficial to keep NK cells active in vivo, which can enhance the immunotherapy efficacy of orthotopic hepatocellular carcinoma (Figure 3).^[^ ^28^ ^]^

## 3 QUANTITATIVE PHOTOACOUSTIC NANOPROBE

Photoacoustic (PA) imaging is a biomedical imaging modality upon the laser‐generated ultrasound, which integrates the optical and ultrasonic technologies.^[^ ^29^ ^]^ Although the photoacoustic effect was early reported at 1880s,^[^ ^30^ ^]^ the employment of PA imaging in biomedical field had not been developed until the past two decades.

PA imaging relies on the absorption of visible or near infrared light by specific light absorbing probes to excite ultrasound signals for detection.^[^ ^31^ ^]^ Similar with the optical imaging, PA imaging possesses high spectral specificity to visualize the anatomical structures of tissues. More importantly, through converting the optical signals into acoustic signals, the tissue penetration depth of PA imaging can be largely boosted.^[^ ^32^ ^]^ In addition, by avoiding the optical scattering of tissues, the spatial resolution of PA imaging can also be improved in comparison with conventional optical imaging.^[^ ^33^ ^]^

On the basis of the above advantages of the PA imaging, in recent years, a series of acidic‐, protease‐, and GSH‐activatable PA imaging nanoprobes have been exploited.^[^ ^18^, ^31^, ^34^ ^]^ For example, Liu et al. synthesized a type of bimetallic oxide MnMoO_X_ nanorods. Such MnMoO_X_ nanorods has no NIR absorption in the absence of GSH, but can exhibit strong NIR absorbance under the presence of GSH, allowing for the responsive detection of GSH with PA imaging. Through making use of this property, these nanorods can serve as smart PA imaging nanoprobes for intratumoral GSH imaging in vivo.^[^ ^35^ ^]^

However, similar with the optical imaging, although the activable PA imaging nanoprobes can specifically respond to the stimuli inside the tumour to generate a stronger PA signal, it is difficult to quantitatively measure the content of stimuli because this PA signal is also affected by the local concentration of the nanoprobes.^[^ ^18a^ ^]^ In this context, by analogy with the optical imaging, the ratiometric stratagem can also be employed in PA imaging based on the activated PA signal and reference PA signal derived from a pair of contrast moieties, which can be further employed to realize the quantitative detect the biomarkers of solid tumours.^[^ ^36^ ^]^

**FIGURE 4:**
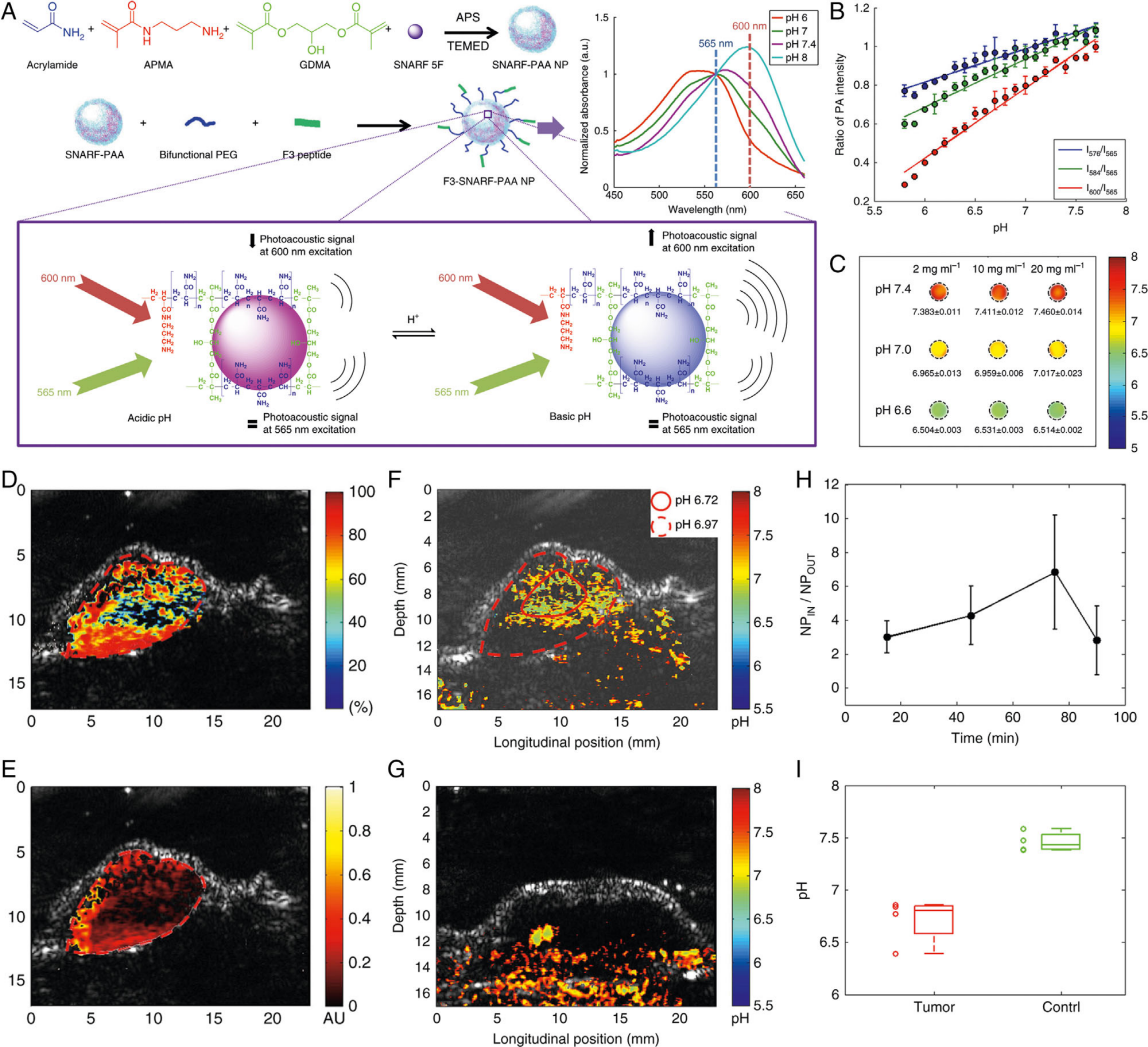
The work principle of SNARF‐PAA nanoparticles and the quantitatively imaging of pH levels and the hemodynamic properties across the entire tumour in vivo. (A) SNARF‐5F encapsulated polyacrylamide‐based nanoparticle (SNARF‐PAA nanoparticle) synthesis and pH‐sensing scheme. (B) Measured PA signal amplitude ratios between the three wavelengths and the isosbestic point (i.e., 576 nm/565 nm, 584 nm/565 nm, and 600 nm/565 nm) from pH 5.8−7.7 with 0.1 pH interval (*n*  =  3, error bars represent standard deviations). (C) Quantitative pH images of phantoms containing different concentrations (2, 10 and 20 mg mL^‐1^) of SNARF‐PAA nanoparticles at different pH levels (pH 6.6, 7.0 and 7.4). PA image showing the spatially distributed (D) haemoglobin oxygen saturation and (E) total haemoglobin concentration in the tumour area at 75 min after injection. (F) PA pH image of a tumour. The pH in the centre area and the peripheral areas are averaged, respectively. (G) Example PA pH image of a normal thigh, showing relatively higher pH. (H) The analysis of the SNARF‐PAA nanoparticles accumulation in the tumours at different time points after systemic injection. (I) The boxplot showing the pH levels in tumours (*n*  =  4) versus the pH levels in normal thigh (*n*  =  4), as quantified from PA pH images. Reproduced with permission.^[^ ^38^ ^]^ Copyright 2017, Nature Publishing Group.

In 2015, Liu et al. reported a pH‐responsive nanoprobe, known as C–HSA–BPOx–IR825, for in vivo quantitative pH imaging. The nanoprobe was fabricated through the cross‐linking of two different types of NIR dyes, that is, benzo[a]phenoxazine (BPOx) and IR825, and human serum albumin (HSA). As a pH‐responsive dye, BPOx can act as a pH indicator in both photoacoustic and fluorescence imaging. In contrast, IR825 possesses the pH‐independent absorbance and fluorescence, which can be served as the internal reference. Compared with the fluorescent pH imaging which is largely restricted by the tissue depth, the ratiometric PA imaging is relatively independent to the tissue depth, which allows the quantitative pH detection of the deeper solid tumours.^[^ ^37^ ^]^ Three years later, Wang et al. also proposed a strategy for quantitatively visualizing the intratumoural pH in vivo using ratiometric PA imaging. In detail, they constructed pH sensitive nanosonophores through encapsulating the SNARF‐5F, a kind of commercially used optical pH indicator, inside the polyacrylamide nanoparticles, known as SNARF‐PAA nanoparticles. Interestingly, the emission wavelength of SNARF‐5F exhibits a pH‐dependent variation, making it possible for the pH quantitative detection through the ratiometric analysis of fluorescent intensities of two emission wavelengths. The nanoparticle‐based systems can not only prevent the SNARF‐5F from interfering by the surrounding biomolecules, but also endow the resultant pH nanoindicator with both cancer specific targeting and immune escaping capabilities. Through spectral unmixing methods, the PA signal contributions of SNARF‐PAA nanoparticles and the haemoglobin can be well separated, thereby providing more precise quantitative pH value within the solid tumour through calibration. In addition, the spatial distribution of total haemoglobin and the haemoglobin oxygen saturation were also mapped through PA imaging. Therefore, not only the pH values of tumour, but also the hemodynamic properties inside the tumour sites could be quantitatively detected through this PA molecular imaging strategy (Figure 4).^[^ ^38^ ^]^

**FIGURE 5:**
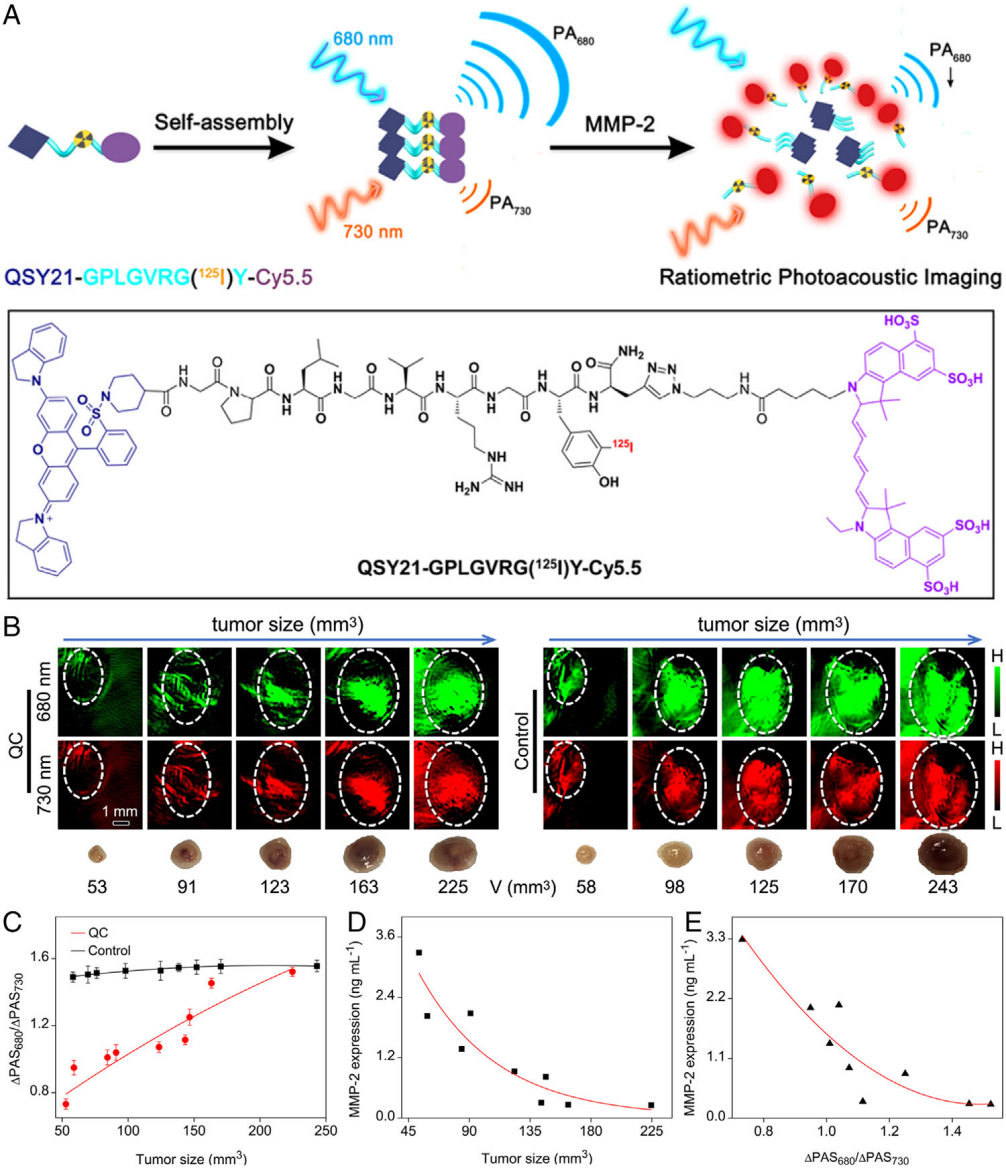
The work principle and structure of the QC probe, together with the quantitative detection of MMP‐2 activity through fluorescence/photoacoustic imaging in vivo. (A) The schematic illustration of QC probe for quantitatively detecting MMP‐2 activity through fluorescence/photoacoustic imaging. (B) PA images of 4T1 tumours of different sizes in vivo, recorded 2 h post injection of the QC probe or its control through 680 and 730 nm channels, together with the photographs of the corresponding tumours harvested right after the PA imaging. Tumour regions are delineated by white dotted circles in the PA images. (C) Ratiometric signal ΔPAS_680_/ΔPAS_730_ against the tumour size. (D, E) MMP‐2 expression levels determined (D) through the conventional tumour‐size‐dependent method and (E) non‐invasively through the ΔPAS_680_/ΔPAS_730_ signal. Reproduced with permission.^[^ ^39^ ^]^ Copyright 2019, American Chemical Society.

Apart from pH, MMP can also be quantitively detected through PA imaging. In 2019, Gao et al. reported a kind of activatable PA probe, which can be adopted for quantitatively imaging the activities of matrix metalloproteinases in vivo. In detail, Cy5.5 and the quencher QSY21 were conjugated by an MMP‐2 substrate peptide, named as QC probe. In water solution, the QC probe that soluble in organic solvent would form nanostructure spontaneously. Interestingly, after the peptide was cut by the MMP‐2 in tumour microenvironment, the aggregation state of QC probes was changed, resulting in an MMP‐2‐concentration‐dependent fluorescent emission. More importantly, this activable assembly will lead to a strong MMP‐2‐responsive absorption band at ≈680 nm, meanwhile the absorption of probe at ≈730 nm was independent to the variation of MMP‐2 concentration. On this basis, the activable PA signal of QD probe at 680 nm (PAS_680_) can be used to visualize the MMP‐2 level in solid tumours, while the PA signal at 730 nm (PAS_730_) can be served as the internal reference. This ratiometric PA system makes it possible to quantitative detect the MMP‐2 expression in the microenvironment of 4T1 solid tumours with different sizes (Figure 5).^[^ ^39^ ^]^ Through using the similar stratagem, they also developed another ratiometric PA probe, which is composed of a hemicyanine fluorophore, a caspase‐3 substrate peptide sequence DEVD, and a tumour‐targeted cyclic RGD peptide sequence. This nanoprobe can quantitatively detect the caspase‐3 activity in the solid tumours in vivo. Upon the cleavage with activated caspase‐3, the dye NH_2_‐Cy‐RGD was generated and self‐assemble to enhance NIR fluorescence signal, and the ratiometric values of PA_710_/PA_680_ is dependent to the concentration of caspase 3, making it possible for quantitatively visualize the caspase‐3 activity with optical and PA imaging.^[^ ^40^ ^]^

Very recently, Shi et al. developed a nanoplatform with H_2_S‐response and consumption abilities, which can be used to quantitatively image and deplete the excessive H_2_S of solid tumours, for realizing colorectal cancer theranostics. In detail, the nanoprobe ZNNPs and folic acid modified ZNNPs (ZNNPs@FA) can be obtained through encapsulating the H_2_S‐responsive hydrophobic NIR‐II fluorophore ZM1068‐NB with biocompatible and amphiphilic polymers mPEG_5000_‐PCL_3000_ or mPEG_5000_‐PCL_3000_‐FA. Such nanoprobes can react with the endogenous H_2_S to specifically convert NIR emission from 1070 to 720 nm. More importantly, the PA signal under 900 nm (PAS_900_) excitation can be gradually decreased against the increasing H_2_S content, meanwhile the PA signal recorded under 680 (PAS_680_) is almost constant with different H_2_S concentration. Through the correlation between PAS_680_/PAS_900_ values and H_2_S concentration, the H_2_S content in acute hepatotoxicity, brain haemorrhage, and colorectal cancer of mice models can be quantitatively detected. In addition, this nanoprobe could also realize the colorectal tumour treatment through H_2_S depletion and photodynamic effects.^[^ ^41^ ^]^

## 4 QUANTITATIVE MRI NANOPROBE

Magnetic resonance imaging (MRI) technology, which is based on the imaging of the relaxation of nuclear magnetic resonance (NMR) signal of nuclei with an odd number of neutrons or protons. With respect to the in vivo imaging, the hydrogen nucleus, which possesses only one single proton, is the most abundant element in the organism. The spin motion and orbital motion of the proton in the hydrogen nucleus result in the magnetic moment of the hydrogen nucleus. Therefore, hydrogen nuclei are the most commonly used imaging nuclei in MRI research. In an external magnetic field, after the radio frequency (RF) pulse is applied and removed, the electromagnetic wave generated during relaxation of magnetic moment can be received and converted into MRI signals.^[^ ^42^ ^]^ Up to now, MRI technology has been served as one of the most powerful imaging technologies widely used for non‐invasive diagnosis of a variety of diseases in clinical practice.^[^ ^43^ ^]^ Although the aforementioned optical and PA imaging technologies are appropriate for quick acquisition of molecular information from the tumour regions in real time, both of these two imaging technologies are still constrained by tissue penetration depth. In this context, MRI with infinite tissue penetration depth, excellent spatial resolution, and high soft tissue contrast, has more practical clinical application value for development.^[^ ^44^ ^]^

Nevertheless, although the MRI can provide high resolution anatomical images of different organs, this contrast is not enough for clearly distinguishing the solid tumours and their surrounding normal tissues.^[^ ^45^ ^]^ The contrast agents, which can shorten the longitudinal or transverse relaxation time of water protons in tissues, were developed to enhanced image quality of MRI.^[^ ^46^ ^]^ Particularly, in recent years, nanoparticle‐based MRI contrast agents have provided promising new platforms to further boost the resolution and sensitivity of MRI for various biomedical applications.^[^ ^47^ ^]^ Through constructing the sophisticated smart MRI nanoprobes, the quantitative visualization of tumour biomarkers can be successfully realized.

### 4.1 Activatable *T*_1_ nanoprobe

As known, *T*_1_ contrast agent can shorten the longitudinal relaxation time of the surrounding water protons, which brighten the *T*_1_‐weighted images.^[^ ^48^ ^]^ In comparison with the conventional nonspecific *T*_1_ contrast agent, the activatable *T*_1_ nanoprobe can specifically generate a brighter signal only within the region of interest, triggered by the specific tumour marker.^[^ ^49^ ^]^ This property could be beneficial in high‐sensitive cancer diagnosis and, importantly, provide a feasible approach to quantitatively detect the stimuli‐related tumour biomarkers.

In 2016, Mi et al. doped paramagnetic Mn^2+^ ion inside the pH responsive calcium phosphate (CaP) nanoparticles. After the nanoparticles accumulated in the solid tumour, the CaP would be degraded by the lower pH of the tumour microenvironment, and the Mn^2+^ can be released outside the nanoparticles to bind with surrounding proteins and interact with water protons, thereby amplifying the *T*_1_ MRI performance. Based on this activation mechanism, the heterogenous *T*_1_ contrast within tumour region in the nanoparticle‐enhanced *T*_1_‐weighted image successfully map the pH distribution in solid tumour. Furthermore, considering that the acidic interstitial pH can be mainly attributed to the lactate accumulation, which is the result of tissue hypoxia. The nanoparticle‐based MRI can also indirectly visualize the lactate distribution and hypoxia region of the solid tumour, which has been confirmed by histological analysis and chemical‐shift imaging.^[^ ^50^ ^]^ Recently, through using this smart nanoprobe, they accomplished the accurate detection and the therapeutic effect prediction of pancreatic tumours via sensing the tumour acidosis and real‐time observing the hypoxic conditions in tumours.^[^ ^51^ ^]^ In 2018, Zhou et al. designed a doxorubicin (DOX)‐loaded Co_3_O_4_ nanoprism, which can respond to the excess H^+^ and H_2_O_2_ in the tumour region to release DOX and Co^3+^, resulting in a *T*_2_ to *T*_1_ contrast switch of MRI of tumour. Through the in vivo  *T*_1_/*T*_2_ ratiometric MRI analysis, the tumour contrast has been significantly improved.^[^ ^52^ ^]^ In 2019, Gao et al. constructed an activable *T*_1_ nanoprobes on the basis of the acidic pH‐triggered Fe^3+^ release. In detail, an upconversion nanoparticle was employed as a core for GA‐Fe(III) complex to grow on the surface, and the resultant nanoprobe is named as UCNP@GA‐Fe(III). Due to the surface unsaturated coordination structure of GA‐Fe(III), Fe^3+^ in the surface of probe could be readily released in response to the lower pH in the tumour microenvironment, thereby activating the *T*_1_ MRI signals. Interestingly, the upconversion luminescence at different wavelengths of nanoprobes can be varied during this Fe^3+^ release process, therefore, the upconversion luminescence can also be employed for quantitatively visualizing the Fe^3+^ release of nanoprobe in vivo.^[^ ^53^ ^]^

The above studies strongly confirm that the activatable *T*_1_ nanoprobes can sense the pathological biomarkers of solid tumours. However, the intrinsic *T*_1_ background of tumour tissue and the passive distribution of nanoprobes still largely limited the precise and quantitative detection of these molecules.

To further explore the possibility of quantitative imaging with *T*_1_ activated nanoprobe, Cheon et al. introduced distance‐dependent magnetic resonance tuning (MRET) principle, which can be served as a feasible strategy for the construction of stimuli‐triggered *T*_1_ enhanced nanoprobe. In a MRET system, a superparamagnetic ‘quencher’ and a paramagnetic ‘enhancer’ was conjugated through an activable linker. At this state, the spin fluctuation of the paramagnetic enhancer is slow, and the water proton relaxation is not enhanced, leading to a quenched *T*_1_ signal. In contrast, after the linker was activated to separate the enhancer and quencher, the enhancer has quick electron‐spin fluctuation to accelerate the relaxation of water protons, leading to a soaring *T*_1_ signal.^[^ ^54^ ^]^ Theoretically speaking, through making use of this principle, the activable *T*_1_ probes can be constructed choosing appropriate activable linkers which can modulate the distance between quencher and enhancer in response to a wide range of stimuli from tumour biomarkers such as pH, enzymes etc. More importantly, because the enhancement of the longitudinal relaxation rate (*R*_1_) of the region of interest can only be attributed to the increase of the concentration of the enhancer released by the activated probe, it can be considered that Δ*R*_1_ is proportional to the biomarker concentration. In this context, the linear correlation between the biomarker concentrations and the Δ*R*_1_ can be used to quantitatively detect the biomarker concentration.^[^ ^55^ ^]^

As a proof of concept, Cheon et al. constructed a MRET probe through covalently conjugating the Gd‐DOTA (paramagnetic enhancers) on the 12 nm Zn_0.4_Fe_2.6_O_4_ nanoparticle (superparamagnetic quencher) through MMP‐2 substrate peptide (activable linker), for MMP‐2 quantitative detection. Expectedly, the *T*_1_ signal of probe solutions increased linearly when the concentration of MMP‐2 enzyme raised from 12.5 to 125 ng mL^‐1^. In the in vivo experiments, the strong *T*_1_ signal appeared in the tumour region after the medication of MRET probe. In contrast, the *T*_1_ signal enhancement of tumour received MMP‐2 inhibtor is inconspicuous under the same administration dose of probe. More importantly, the Δ*R*_1_ of different tumours were calculated, and the real MMP‐2 content of these tumour tissues were also detected by western blot analysis. As a result, the correlation between Δ*R*_1_ and MMP‐2 contents reveals that the MMP‐2 content inside the tumours can be successfully reflected by the variation of *T*_1_ signals. These results demonstrated that the MRET imaging strategy makes it possible to quantitatively image the tumour biomarkers in vivo without the constraint of the tissue penetration depth.

Based on the MRET system, Bu et al. developed a GSH‐responsive susceptibility weighted imaging (SWI) nanoprobes, for realizing the in situ detection of GSH contents inside the solid tumours in vivo. In detail, SWI nanoprobes were constructed by linking the Fe_3_O_4_ nanoparticle and CoFe_2_O_4_ nanoparticles with cystamine linker. When the disulfide bonds inside the nanoprobes are cleaved by excessive GSH in solid tumour, the enlarged distance between Fe_3_O_4_ and CoFe_2_O_4_ nanoparticles would lead to magnetic exchange coupling effect decrease. The variation SWI signals in this process can be used for high‐sensitive in vivo GSH concentration detection.^[^ ^56^ ^]^ In another work, Hou et al. constructed a *T*_1_/*T*_2_ dual‐modality MR Imaging theranostic nanoprobe on the basis of protein corona coated Fe_3_O_4_ nanoparticles and biomineralization inside the corona. Specifically, the bovine serum albumin (BSA) molecules adsorbed on the carboxylic Fe_3_O_4_ nanoparticles were served as the matrixes for biomimetically mineralizing Fe^3+^ ion with tannic acid (TA). The TA‐Fe(III) biominerals on the surface of nanoprobes can serve as the photothermal therapy agents and, more importantly, interact with unsaturated transferrin in plasma to form a “hybrid” corona, which endows the nanoprobes with tumour targeting ability. In this design concept, when Fe^3+^ ions are confined in the BSA‐TAFe^III^ biominerals, the *T*_1_ effect is suppressed by the Fe_3_O_4_ nanoprobes according to MRET effect. In contrast, in the acidic region of solid tumours, the protonation of phenol hydroxyl groups would result in the release of Fe^3+^, which can boost the *T*_1_ MRI signals by breaking the MRET interaction.^[^ ^57^ ^]^ Considering that the *T*_2_ signal derived from the Fe_3_O_4_ nanoparticles is independent to pH, the heterogeneous distribution of *T*_1_ signal in tumour tissues has the potential to map the pH in tumour region after calibration.

**FIGURE 6:**
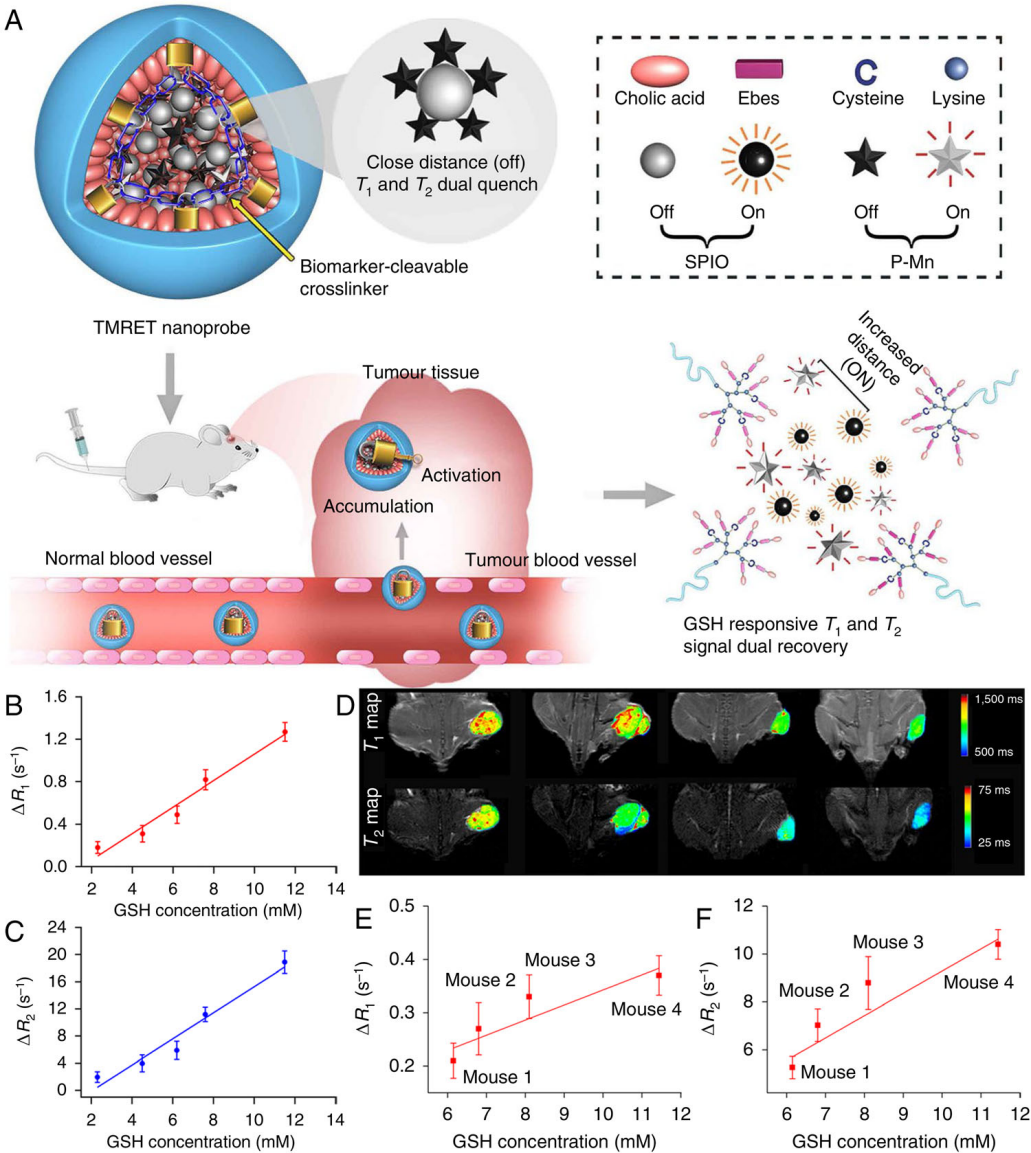
The structure and work principle of TMRET nanoprobe, together with the quantitative MRI of GSH concentration of tumours in vivo. (A) Schematic illustration of the TMRET nanotechnology. Mn^2+^ conjugated to pheophorbide serves as both an ‘enhancer’ in the *T*_1_ MRI signal and a ‘quencher’ in the *T*_2_ MRI signal, whereas the SPIO nanoparticle acts as an ‘enhancer’ in the *T*_2_ MRI signal and a ‘quencher’ in the *T*_1_ MRI signal. (B, C) Δ*R*_1_ and Δ*R*_2_ of in PC‐3 cells treated with various concentrations of the GSH inhibitor (n = 3). Δ*R*_1_ and Δ*R*_2_ of the TMRET nanoprobe were measured to be 0.18, 0.31, 0.49, 0.82 and 1.27 s^−1^ (Δ*R*_1_) and 1.96, 3.97, 5.89, 11.20 and 18.90 s^−1^(Δ*R*_2_), respectively. The GSH concentrations in cells were measured by using thiol tracker violet (GSH detection reagent). Reproduced with permission.^[^ ^59^ ^]^ Copyright 2020, Nature Publishing Group.

In addition to the construction of the activatable *T*_1_ MRI nanoprobe, MRET system can also be employed to design *T*_1_ and *T*_2_ double‐activated probes. In comparison with the above *T*_1_ MRI nanoprobe, the double‐activated probes combine the advantages of two MRI sequences, possessing the potential to enhance the imaging sensitivity, therefore making the quantitative analysis more accurate. For example, Zhang et al. designed a *T*_1_ and *T*_2_‐dual‐mode MRI nanoswitch. In detail, the Mn‐doped silica‐coated Fe_3_O_4_ nanoparticles with yolk‐shell nanostructures were synthesized. In addition, the platelet‐derived growth factor molecules were further conjugated to endow the yolk‐shell nanostructures with tumour targeting ability. On the one hand, owing to the MRET effect, the *T*_1_ effect of Mn^2+^ will be effectively quenched by the Fe_3_O_4_ nanoprticles. On the other hand, when Fe_3_O_4_ and Mn‐doped silica are in direct contact, Mn^2+^ may disrupt the magnetic moments, leading to the quenching of the *T*_2_ effect. In the acidic tumour microenvironment with rich GSH, the Mn─O bond in the Mn‐doped silica shell would break, resulting in the shell collapse and the release of Mn^2+^. Since the reduction of the mutual interference between Mn^2+^ and Fe_3_O_4_, the dual‐quenching effects will be relieved, thus generating the enhanced *T*_1_ and *T*_2_ MRI signals.^[^ ^58^ ^]^ For realizing the quantitative imaging, Li et al. developed a two‐way magnetic resonance tuning (TMRET) nanoprobe with dually activatable *T*_1_ and *T*_2_ signals. TMRET nanoprobe was established by simultaneous encapsulation of a two‐way MRET pair, that is, a chelate of pheophorbide a–Mn^2+^ (P–Mn) and a superparamagnetic iron oxide (SPIO) nanoparticle, into a responsive micelle. When this TMRET pair is locked inside the micelle, both *T*_1_ and *T*_2_ signals would be quenched. In contrast, after activated by the tumour stimuli, the micelle would be collapsed to release the Mn^2+^ and SPIO, thereby dually enhancing both *T*_1_ and *T*_2_ signals through the increased distance between Mn^2+^ and SPIO. Such activable TMRET system allows the precise and quantitative detection of tumour biomarkers. As a proof of concept, through using the reversible disulfide cross‐linked micelles (DCM) that can be triggered to release the payloads under the stimulation of tumoral GSH, they constructed the GSH responsive TMRET nanotechnology platform DCM@P–Mn–SPIO for the noninvasive and quantitative detection of GSH in tumour regions. Through this TMRET nanoplatform, the relationship between the intratumoural GSH content and the *T*_1_/*T*_2_ signals of TMRET nanoprobe were quantitatively analyzed. After intravenous injection of the nanoprobes into the tumour‐bearing mice, both the Δ*R*_1_ and Δ*R*_2_ of tumour region increased linearly with the intratumoural GSH contents, implying that such nanoprobes may be useful for the in vivo quantitative visualization of GSH (Figure 6).^[^ ^59^ ^]^ More recently, Wang et al. designed and constructed a dual‐ratiometric magnetic resonance tunable nanoprobe. Specifically, Gd and transferrin (Tf) were fabricated through biomineralization‐like process. The resultant Gd‐Tf, together with SPIO, were encapsulated into a pH‐responsive polymer micelle. Under neutral environment, the *T*_1_ and *T*_2_ effect of nanoprobe remained at “off” state owing to the distance‐dependent quenching effect between Gd and SPIO. However, when the probe is situated within an acidic environment, the nanoprobe would disassemble to switch to “on” state, leading to the both recovery of *T*_1_ and *T*_2_ MRI signals. In this context, the *T*_1_‐*T*_2_ dually activatable nanoprobe possess the potential to quantitatively visualize the pathophysiological information in vitro and in vivo.^[^ ^60^ ^]^

### 4.2 Responsive aggregation nanoprobe

Iron oxide‐based nanoparticle is a category of MRI contrast agents that has been widely employed in clinical and pre‐clinical trials owing to a combination of favourable superparamagnetic properties, biocompatibility, and surface properties.^[^ ^61^ ^]^ Until now, it is still widely accepted that iron oxide nanoparticle contrast agent would be a better alternative of Gd‐based contrast agent, especially for the patients with chronic kidney disease, because the nanoparticles can bypass the kidney filtration and does not contain toxic free Gd.^[^ ^62^ ^]^ In the past two decades, this class of nanoparticles has been employed to establish different kinds of sensitive nanoprobes for accurate cancer diagnosis.^[^ ^63^ ^]^ Interestingly, the longitudinal/transverse relaxivity of superparamagnetic iron oxide nanoparticles could be tuned through varying their particle diameter or aggregation status.^[^ ^64^ ^]^ The smaller iron oxide nanoparticles tend to exhibit a paramagnetic property owing to the 5 unpaired electrons of Fe^3+^ on the surface of nanoparticles, which can served as *T*_1_ MRI contrast agents. In comparison, when the diameter of iron oxide nanoparticles increased, or the small nanoparticles aggregate to form the large agglomerates, the magnetic moment would be largely improved due to the enhancement of volume magnetic anisotropy. Therefore, the large superparamagnetic iron oxide nanoparticles or the particle agglomerates can be regarded as better *T*_2_ MRI contrast agents.^[^ ^65^ ^]^

Based on this principle, in 2016, Liang et al. design a type of caspase 3‐activated nanoprobe. Owing to the responsive surface ligands, the Fe_3_O_4_‐based nanoprobe can aggregate to generate a stronger *T*_2_ signal under the triggering of caspase‐3 in the apoptotic cells in vitro and in vivo, therefore, this nanoprobes could be used to visualize the caspase‐3 level in the solid tumour. Through this MRI strategy, the nanoprobes have the potential to be employed in the evaluation of the chemotherapeutic outcome of cancer.^[^ ^66^ ^]^ In addition to the *T*_2_ enhanced nanoprobe, the *T*_1_–*T*_2_ switchable nanoprobes have been also constructed through using similar strategies. For example, Mao et al. synthesized the sub‐5 nm ultrafine oligosaccharide‐coated iron oxide nanoparticles as the *T*_1_–*T*_2_ switchable MRI contrast agent. The nanoparticles can extravasate deep in the solid tumor and, more importantly, generate large aggregates in the acidic microenvironment. In the in vivo MRI studies, these nanoparticles exhibited bright *T*_1_ signal at 1 h post‐injection, followed by the enhanced dark *T*_2_ signal after 24 h. In another study, Chen et al. synthesized the hyaluronic acid (HA)‐coated Fe_3_O_4_ nanoparticles to target the tumour cells and realize the in vivo switchable *T*_1_‐ to *T*_2_‐weighted MRI by the aggregation of Fe_3_O_4_ in the tumour microenvironment after the surface HA was degraded by the abundant hyaluronidase (HAase) in acidic condition.^[^ ^67^ ^]^ The similar strategy has been also employed to construct the *T*_1_–*T*_2_ switchable MRI nanoprobe for the non‐invasive identification of vulnerable atherosclerotic plaques.^[^ ^68^ ^]^ Near recently, Pei et al. synthesized the nitroimidazole derivatives and cysteine conjugated extremely small iron oxide nanoparticles, which can form nanocluster in the hypoxic tumour microenvironment, realizing the *T*_1_–*T*_2_ switchable MRI of tumour.^[^ ^69^ ^]^ In addition to the *T*_1_–*T*_2_ switchable MRI nanoprobes, the *T*_2_–*T*_1_ switchable nanoprobe can also be constructed based on the disaggregate of the superparamagnetic nanoparticles. Ling et al. designed a pH‐sensitive iron oxide nanoparticle assemblies cross‐linked by aldehyde derivative crosslinkers. In the acidic tumour region, the hydrazone bonds would be cleaved, leading to the collapse of assemblies. The resultant dispersed iron oxide nanoparticles can significantly enhance the *T*_1_ effect to realize the sensitive tumour detection.^[^ ^70^ ^]^ Through the similar strategy, Yang et al. also designed and synthesized the assembly of ultra‐small Fe_3_O_4_ nanoparticles crosslinked by cystamine, which can realize the *T*_2_–*T*_1_ switchable imaging in tumour sites through the cleavage of disulfide bond induced by excess GSH.^[^ ^71^ ^]^

**FIGURE 7:**
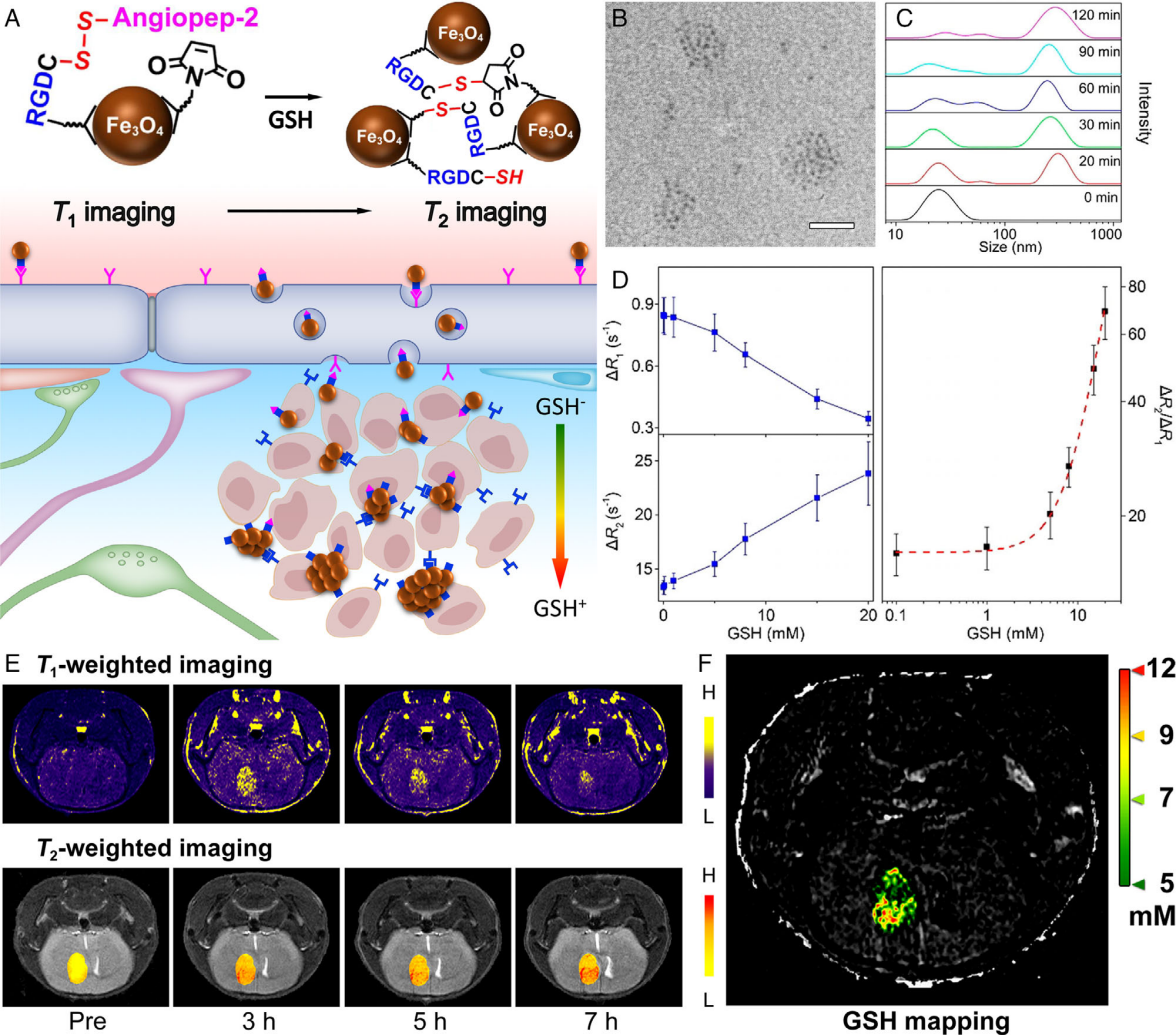
The structure, characterization, and work principle of *T*_1_/*T*_2_ interlocked nanoprobe, together with the quantitatively mapping of GSH in intracranial tumour in vivo. (A) Schematic drawings to show the mechanism of the GSH‐induced agglomeration of the nanoprobes and MRI quantitative imaging principle. (B) TEM images of nanoprobes after treatment with GSH (the embedded scale bar corresponds to 30 nm). (C) Temporal hydrodynamic size profiles of nanoprobes in reactions to GSH treatment. (D) GSH concentration dependent Δ*R*_1_, Δ*R*_2_, and Δ*R*_2_/Δ*R*_1_ for nanoprobe after being incubated with different concentrations of GSH, together with a theoretical fitting (red dashed line). (E) *T*_1_‐ and *T*_2_‐weighted MR images of mice bearing orthotopic U87MG glioblastoma xenografts acquired before and at different time points after the intravenous injections of nanoprobes. (F) Quantitative mapping of GSH according to imaging data acquired at 7 h post‐injection through the correlation between Δ*R*_2_/Δ*R*_1_ and GSH concentration in vivo. Reproduced with permission.^[^ ^74^ ^]^ Copyright 2021, Wiley‐VCH.

These studies strongly confirmed the feasibility of the iron oxide responsive aggregation‐based MRI system in specifically diagnosis of tumour biomarkers. Nevertheless, owing to the intrinsic *T*_2_ signal of the tumour regions, it may be difficult to quantitatively visualize the biomarkers in vivo. In 2017, Gao et al. developed a smart nanoprobe for tumour MRI precise diagnosis. The nanoprobe was constructed through modifying Fe_3_O_4_ nanoparticles with disulfide bond‐contained peptides. In the GSH‐rich regions of solid tumour, the disulfide bond on nanoprobes will be cleaved to generate thiol groups, which can then crosslink with the free maleimide groups on the surface of adjacent nanoprobes to form the aggregates in situ, thereby significantly enhancing the *T*_2_ signal in high GSH level regions. Through this strategy, they realize the high‐sensitive MRI diagnosis of solid tumours.^[^ ^72^ ^]^ On this basis, they attempted to quantitatively detect GSH level in solid tumours. In 2021, with the similar responsive design, Zhang et al. modified the disulfide bond‐contained BBB shuttle peptides onto the surface of ultrasmall Fe_3_O_4_ nanoparticles to construct GSH responsive nanoprobe. Owing to the tiny diameter of the core Fe_3_O_4_ nanoparticle, the monodisperse nanoprobes exhibited a strong *T*_1_ MRI performance. After these monodisperse nanoprobes cross blood‐brain barrier and enter the tumour region with the help of BBB shuttle peptides, the heterogeneous spatial distribution of GSH in the intracranial solid tumour will lead to aggregation of nanoprobes with vary degrees, thereby generating *T*_2_ MRI signals with different intensities and weakening the original *T*_1_ MRI signals correspondingly (Figure 7A–C). Through theoretical derivation of the aggregation process, and the mathematical analysis of the correlation between the aggregation and the interlocked changes of Δ*R*_1_ and Δ*R*_2_, the local GSH content can be quantitatively calculated by the responsive MRI signals (Figure 7D). This quantitative correlation can be further used for noninvasively visualizing the heterogeneous GSH content inside a tiny intracranial tumour with the size of 2.4 mm × 1.6 mm in vivo (Figure 7E,F).^[^ ^73^ ^]^

## 5 CONCLUSION AND OUTLOOK

In this review, the state of art of design and application of quantitative molecular imaging nanoprobes for cancer pathological molecules visualization were summarized. The imaging type, quantitative sensitivity, and optimal application scenario of representative molecular imaging probes have been listed in Table 1. Among them, the studies of nanoprobe‐based quantitative optical imaging are the most reported, which displays excellent performance, including high sensitivity and real‐time feedback. Additionally, due to the portable and movable characteristics of the light source, the use of optical imaging with low‐cost imaging facilities will be more convenient for the cancer patients. However, the accuracy of quantitative results may be largely disturbed by the tissue penetration depth of excitation and emission light. In this context, the quantitative optical imaging can be employed during the surgical resection of tumours to assist surgeons to distinguish the tumour boundaries and the tumour metastases at the sentinel lymph nodes, thereby ensuring the complete resection of tumour and avoiding the recurrence.^[^ ^74^ ^]^ In comparison, the nanoprobe‐based quantitative PA imaging, which depends on the excitation of light and the detection of ultrasonic signals, exhibits a deeper tissue penetration depth and resolution for better quantification of the deeper tumours than the traditional optical imaging. Thus, the quantitative PA imaging may better suitable for the detection and biopsy/surgery guidance of superficial cancers, such as breast cancer, skin cancer, or cancers at the reproductive and urological systems.^[^ ^33b^ ^]^ In addition, the nanoprobe‐based quantitative MRI enjoys the infinite tissue penetration depth, excellent in vivo spatial resolution and tissue discernment ability, which can provide tomographic biomolecular distribution images of deep solid tumour. However, the long scan time for different sequences makes it difficult for MRI to provide real‐time feedback images like optical and PA imaging. In addition, the sophisticated principle and complicated parameters of MRI makes the quantitative imaging a great challenge. Therefore, in the following studies, the quantitative MRI may be mainly used in the diagnosis of small solid tumours located in the deep tissues, or in the fundamental research of in vivo tumour molecular biology.

**TABLE 1:** Summary of imaging type, quantitative sensitivity, and optimal application scenario of representative molecular imaging probes.

Probe	Imaging type	Quantitative sensitivity	Optimal application scenario	Ref.
^64^Cu‐NOTA‐YY146	PET/CT	——	CD146‐targeted orthotopic and metastatic breast cancer imaging	^[^ ^12a^ ^]^
^89^Zr‐Df−YY146	PET	——	CD146‐targeted malignant brain tumours imaging	^[^ ^12b^ ^]^
^89^Zr‐Df‐YY146‐ZW800	PET/NIR OI	——	CD146‐targeted molecular imaging in liver malignancies	^[^ ^12c^ ^]^
NaErF_4_@NaYbF_4_@NaYF_4_‐FA	UCL/NIR‐II OI	——	Folate receptor distribution mapping of intraperitoneal tumours	^[^ ^13a^ ^]^
NaGdF_4_:Yb,Er@NaGdF_4_‐FA	UCL OI	——	Folate targeted visualization of colorectal tumours.	^[^ ^13b^ ^]^
ANNA	Ratiometric OI	pH mapping (6.36–8.13) in vitro	pH responsive imaging in living cells	^[^ ^19^ ^]^
TAT‐ANNA	Ratiometric OI	pH mapping (4.84‐8.23) in vitro	Noninvasively evaluation of chemotherapy efficacy	^[^ ^20^ ^]^
ANNA/Fe_3_O_4_	Ratiometric OI	Tumour pH mapping (6.6–7.6) in vivo	Quantitatively mapping the pH values in tumour	^[^ ^21^ ^]^
Fe_3_O_4_ /ANNA/FA/Cy5.5	Ratiometric OI	Tumour MMP‐9 mapping (4.3–6.8 ng mL^−1^) and pH mapping (6.6–7.6) in vivo	Quantitatively mapping the MMP‐9 activity and pH of tumours in vivo	^[^ ^22^ ^]^
Ir‐BTPHSA/CD‐Cy7	Ratiometric OI	Oxygen levels 0−100% ex vivo, 0–21% in vitro,1–14% in vivo	Quantitatively determining the oxygen level in vivo	^[^ ^23^ ^]^
TPFPs	Ratiometric OI	Nitroreductase concentration (0–10 μg mL^−1^, down to 0.142 ng mL^−1^) in vitro	Visualizing endogenous nitroreductase activities in tumour	^[^ ^24^ ^]^
Cy7‐1/PG5‐Cy5@LWHA	Ratiometric OI	Nitroreductase concentration (0–1.5 μg mL^−1^) in vitro	Quantitative tumour hypoxia imaging in vivo	^[^ ^25^ ^]^
TPEF‐GSH	Ratiometric OI	GSH detection (3.125–9.375 mm) in vivo	Quantification and visualization of GSH in pretreated living embryos.	^[^ ^26^ ^]^
DCNP/IR786s	Ratiometric NIR‐II OI	NK cell viability (0–100%) in vitro	Tracking of NK cell viability in vivo	^[^ ^28^ ^]^
MnMoOx‐PEG	PAI/MRI	Generating responsive signals to GSH (0–20 mm, down to 0.5 mm) in vitro	GSH detection of tumour in vivo	^[^ ^35^ ^]^
C‐HSA‐BPOx‐IR825	Ratiometric PAI/OI	Generating responsive signals to pH (4–8) in vitro	pH responsive tumour imaging	^[^ ^37^ ^]^
F3‐SNARF‐PAA	Ratiometric PAI	Generating response signals to pH (5.8–7.8) in vitro	pH responsive tumour imaging	^[^ ^38^ ^]^
QSY21‐GPLGVRGY‐Cy5.5	OI/Ratiometric PAI	MMP‐2 detection (0‐160 ng mL^−1^, down to 0.52 ng mL^−1^) in vitro	Quantitative imaging of MMP‐2 in tumour in vivo	^[^ ^39^ ^]^
AcDEVD‐Cy‐RGD	NIR OI/ Ratiometric PAI	Caspase‐3 detection (0–500 ng mL^−1^, down to 3.4 ng mL^−1^) in vitro	Quantitative imaging of caspase‐3 in breast cancer models in vivo	^[^ ^40^ ^]^
ZNNPs@FA	Ratiometric OI/PAI	NaHS detection of Fluorescence responsiveness (0–500 μm, down to 4.7 μm) and PA responsiveness (0‐500 μm, down to 0.68 μm) in vitro; H_2_S detection of injured brain (≈5.29 μmol g^−1^ protein) in vivo	Quantitative detection and imaging of H_2_S in vivo as well as photodynamic therapy of colorectal tumours.	^[^ ^41^ ^]^
PEGMnCaP	*T*_1_ MRI	Generating response signals to pH (5.0, 6.0, 6.5 , 7.4) in vitro	pH and lactate mapping of tumour in vivo	^[^ ^50^ ^]^
Mn^2+^‐doped CaP	*T*_1_ MRI	Generating response signals to pH (5.0–7.4) and with or without hypoxia in vitro	Imaging of pancreatic tumours in hypoxia condition	^[^ ^51^ ^]^
Co_3_O_4_‐DOX	*T*_1_/*T*_2_ MRI	Generating response signals to H_2_O_2_ (0–50 μm, down to 50 nm) and pH (6.5,7.5) in vitro	H_2_O_2_ and pH responsive tumour diagnosis and combined therapy	^[^ ^52^ ^]^
UCNP@GA‐Fe^III^	*T*_1_ MRI /UCL OI	Generating response signals to pH (3–8) in vitro	Activatable tumour diagnosis and combined therapy	^[^ ^53^ ^]^
Gd‐DOTA/Zn_0.4_Fe_2.6_O_4_/PLGVR	*T*_1_ MRI	Generating response signals to MMP‐2 (12.5–125 ng mL^−1^) in vitro and the amount of MMP‐2 (0.41, 0.63, 0.81, 1.33 μg mL^−1^) in each tumour	Quantitative analysis of MMP‐2 expression level in tumours in vivo	^[^ ^54^ ^]^
Fe_3_O_4_ ‐S‐S‐CoFe_2_O_4_	SWI/*T*_2_ MRI	Generating response signals to GSH (0–6 mm) in vitro	GSH‐triggered tumour imaging in vivo	^[^ ^56^ ^]^
Fe_3_O_4_ @BSA‐TAFe^III^	*T*_1_/*T*_2_ MRI	Generating response signals to H_2_O_2_ (0–10 mm) and pH (4.5, 5.5, 6.5,7.5) in vitro	pH responsive tumour imaging and combined therapy	^[^ ^57^ ^]^
PDGFB‐FMS	*T*_1_/*T*_2_ MRI	Generating response signals to pH (4.5, 5.5, 6.5,7.4) and GSH (0, 5, 10, 20 mm) in vitro	GSH and pH responsive tumour diagnosis	^[^ ^58^ ^]^
DCM@P‐Mn‐SPIO	*T*_1_/*T*_2_ MRI	Generating response signals to GSH (0, 5, 10, 20 mm,) in vitro; Tumour GSH mapping (6.15–11.44 mM) in vivo	Quantitative GSH tumour imaging in vivo	^[^ ^59^ ^]^
PEG@Gd‐Tf/ PEG@SPIO	*T*_1_/*T*_2_ MRI	Generating response signals to pH (5.0, 5.5, 6.8, 7.4) in vitro	pH responsive early tumour pathological imaging in vivo	^[^ ^60^ ^]^
Fe_3_O_4_ @1 NP	*T*_2_ MRI	Generating responsive signals under 1 mm GSH and 5 unit Caspase 3 in vitro	Caspase3 responsive tumour apoptosis imaging	^[^ ^66^ ^]^
Fe_3_O_4_@HA_280_	*T*_1_/*T*_2_ MRI	Generating response signals to pH (4.5, 5.0, 6.0, 7.4) in vitro	pH responsive tumour imaging in vivo	^[^ ^67^ ^]^
IONP‐HP	*T*_1_/*T*_2_ MRI	Generating response to pH (4, 5, 6, 7.4) in vitro	pH responsive vulnerable atherosclerotic plaques imaging in vivo	^[^ ^68^ ^]^
HR‐ESIONPs	*T*_1_/*T*_2_ MRI	Generating response signals to normoxia (≈40 mmHg pO_2_) and hypoxia (less than 10 mmHg pO_2_) in vitro	Hypoxia responsive tumour imaging in vivo	^[^ ^69^ ^]^
ESIONs	*T*_1_/*T*_2_ MRI	Generating response signals to pH (7.5, 5.5) in vitro	pH responsive tumour imaging in vivo	^[^ ^70^ ^]^
Fe_3_O_4_‐DHCA	*T*_1_/*T*_2_ MRI	Generating response signals to GSH (0, 4, 10 mm) in vitro	GSH responsive tumour imaging in vivo	^[^ ^71^ ^]^
^99m^Tc‐labeled Fe_3_O_4_‐RGD‐S‐S‐self‐peptide	*T*_2_ MRI/SPECT/CT	Generating response signals to GSH (0, 0.1, 1, 5, 10 mm) in vitro	GSH responsive tumour imaging in vivo	^[^ ^72^ ^]^
Fe_3_O_4_‐RGD‐S‐S‐Angiopep‐2 peptide	*T*_1_/*T*_2_ MRI	Tumour GSH mapping (0–20 mm) in vivo	Quantitative intratumoural GSH mapping in vivo	^[^ ^73^ ^]^

Apart from the above imaging modalities, nuclear medicine imaging (NMI), mainly including single‐photon emission computed tomography (SPECT) and positron emission tomography (PET) imaging, has been developed as a powerful approach for cancer diagnosis. Through the detection of gamma rays emitted from the radioactive tracers, the diagnostic images can be obtained with high sensitivity and unlimited penetration depth. More importantly, the spatial distribution of radioactive tracers can be quantitatively measured. Different from the above quantitative imaging strategies that quantify tumour biomarkers through the corrected intensities of newly generated signals, NMI directly quantifies the concentration of radioactive tracer itself. In this context, using the radiolabelled metabolism‐related molecules as tracers, the tumour tissues and normal tissues can be distinguished by the metabolic differences. For example, in clinical PET imaging, fluorodeoxyglucose (^18^F‐FDG) has been widely used as the tracer for cancer diagnosis. As a glucose analogue, the tissue accumulation of ^18^F‐FDG can reveal their glucose metabolism level. Due to the vigorous metabolism, most tumour tissues exhibit high uptake of ^18^F‐FDG, which will be recognized as hyperintense regions in PET images. In addition, Because the glutamine can promote the growth of cancer cells, the ^18^F‐(2S,4R)4‐fluoroglutamine (^18^F‐Gln) has been employed to evaluate glutamine metabolism in the tumour microenvironment. For example, in the laboratory researches of tumour PET imaging, subcutaneous MC38 tumours displayed a high ^18^F‐Gln avidity.^[^ ^75^ ^]^ With the development of radiolabelling strategies in recent years, a variety of radiolabelled nanomaterials have been explored to serve as the nano‐tracers for tumour diagnosis, which largely improved the imaging performance compared with traditional NMI. With the quantitative capability of tracers, NMI can serve as an ideal complement when combined with the aforementioned imaging modalities, thereby providing more accurate quantitative analysis of tumour biomarkers. The overview of radiolabelling nanomaterials for cancer diagnosis has been discussed previously, and the detailed information can be found in a recent review paper.^[^ ^76^ ^]^

Considering that each imaging modality possess distinct merits and demerits, one single imaging modality cannot realize the comprehensive detection of tumour, therefore, nanoprobe‐based dual‐modal or multi‐modal imaging strategies has been widely developed for cancer diagnosis in different clinical and pre‐clinical trials.^[^ ^77^ ^]^ Undoubtedly, the integration of the complimentary advantages of different imaging modalities will be a useful strategy to provide more abundant and reliable biomedical details, thereby largely enhancing the precision of the imaging diagnosis of cancers. In this context, the multi‐modalities quantitative molecular nanoprobe is much needed to visually measure the biomarkers of tumour through different imaging modalities.

Additionally, considering that the tumour microenvironment is a dynamic system involving the interaction of multiple physical and biochemical factors, single marker detection may be difficult to provide sufficient information for accurate tumour diagnosis. Simultaneous detection of multiple biomarkers will give a more accurate diagnosis of cancers, which has been commonly performed in the clinical biopsy diagnosis of cancers. In this context, realizing the simultaneous visualization of multiple biomarkers of tumour through quantitative molecular imaging technologies is highly anticipated. Among the above imaging technologies, fluorescence imaging may be a better imaging modality to realize this expectation. In detail, the smart nanoprobes can be designed to possess distinguishable fluorescence resonance energy transfer systems, corresponding to the detection of different tumour biomarkers. On the basis of this strategy, in the past decade, many attempts have been made to simultaneously visualize different tumour biomarkers using nanoprobe‐based fluorescence imaging.^[^ ^78^ ^]^ Nevertheless, most of these imaging studies remain at the cellular level, and the research at the in vivo level is still insufficient, let alone in vivo quantitative detection. Therefore, constructing new quantitative molecular imaging nanoprobes, which possess “logical” response capabilities to multiple biomarkers in tumour microenvironment, will be one of the key points of future research. With these types of nanoprobe, the diagnosis of cancer will be more accurate, and the correlation of different biomarkers during tumour development process in vivo will also be revealed.

In comparison with the conventional targeted nanoprobes and qualitative activated nanoprobes, the quantitative nanoprobes can visualize the accurate levels of pathological molecules in the solid tumours including pH, specific enzyme, redox states, hypoxia etc. This nanoprobe‐based molecular imaging technology can build a bridge between the molecular biological researches of cancer and the clinical practice and development. That is, molecular biology research is responsible for defining quantitative standards and grading strategies for different biomarkers of cancer, while the quantitative imaging can provide the actual values of these parameters in different solid tumours of every individual. For the early primary cancer, the nanoprobe‐based quantitative molecular imaging can visualize the specific molecular expression status in different patients, so as to realize the better selection of individualized, target‐guided tumour treatment. Additionally, according to the quantitative imaging results, the malignancy and classification of tumours can be determined quickly on the early stage. For the cancers in progressive stage, the nanoprobe‐based quantitative molecular imaging can monitor the temporal evolution of the molecular characteristics inside the tumours, which may serve as predictors of the tumour prognosis, and provide more basis for the adjusting the treatment strategies. Overall, the results of such nanoprobe‐based quantitative molecular imaging strategy can serve as the criteria to replace the subjective evaluation of the doctors, which will be helpful for making clinical decisions and greatly reduce the possibility of misdiagnosis.

Nevertheless, despite the satisfactory experiment results of the nanoprobe‐based quantitative imaging in laboratory studies, the actual clinical translation of this strategy still faces great challenges. Firstly, it should be conceded that the design and construct of the sophisticated molecular imaging nanoprobe, as well as developing the new activable strategies for quantitative detection of cancer biomarkers, are very challenging in practice. Researchers still need to find new biomarkers that can be used to establish corresponding quantitative strategies. Secondly, the full impact of different nanoprobes on the body is still unknown, which needs to be systematically investigated through extensive safety studies.^[^ ^79^ ^]^ For instance, whether the long‐term hepatic retention of nanoprobes will increase the liver burden and even lead to irreversible hepatic injury should be carefully assessed.^[^ ^80^ ^]^ Furthermore, owing to the complicated structure of the most quantitative molecular imaging nanoprobes, the large‐scale synthesis with reproducible activated features will be very difficult. At last, the inoculated of implanted solid tumours in rodent animals may be very different from that of humans.^[^ ^81^ ^]^ Thus, the quantitative nanoprobes should be further evaluated on large animal models, such as non‐human primate models, especially for evaluating whether the correlation between the imaging signals and biomarkers concentrations is still valid.

Even so, there is still reason to expect that the nanoprobe‐based quantitative molecular imaging will be translated to become an indispensable component of clinical diagnosis of cancer in the future, which will then allow the suitable and customized treatment strategies of every individual.

## CONFLICT OF INTEREST STATEMENT

The authors declare no conflicts of interest.
